# Detection of the chronic kidney disease using XGBoost classifier and explaining the influence of the attributes on the model using SHAP

**DOI:** 10.1038/s41598-023-33525-0

**Published:** 2023-04-17

**Authors:** Md. Johir Raihan, Md. Al-Masrur Khan, Seong-Hoon Kee, Abdullah-Al Nahid

**Affiliations:** 1 Electronics and Communication Engineering Discipline, Khulna University, Khulna, 9208 Bangladesh; 2 Department of ICT Integrated Ocean Smart Cities Engineering, Dong-A University, Busan, 49315 South Korea

**Keywords:** Diseases, Health care, Medical research

## Abstract

Chronic kidney disease (CKD) is a condition distinguished by structural and functional changes to the kidney over time. Studies show that 10% of adults worldwide are affected by some kind of CKD, resulting in 1.2 million deaths. Recently, CKD has emerged as a leading cause of mortality worldwide, making it necessary to develop a Computer-Aided Diagnostic (CAD) system to diagnose CKD automatically. Machine Learning (ML) based CAD system can be used by a clinician to automatically diagnoses mass people. Since ML models are considered a black box, it is also necessary to expose influential causes behind a model's prediction of a particular output. So that, a doctor can make a more rational decision based on the model's output and analysis of the features influence on the model. In this paper, we have used the XGBoost as the ML classifier to predict whether a patient has CKD or not. Using the XGBoost classifier, we have obtained an accuracy, precision, recall, and F1 score of $99.16{\%}, 100{\%}, 98.68{\%},$ and $99.33{\%},$ respectively using all $24$ features. Furthermore, we have used Biogeography Based Optimization (BBO) algorithm to find an effective subset of the features. The BBO algorithm selected almost half of the initial features. We have obtained an accuracy, precision, recall, and F1 score of $98.33{\%}, 100{\%}, 97.36{\%},$ and $98.67{\%},$ respectively using only 13 features selected by the BBO algorithm. Finally, we have explained the impact of the feature on the ML models using the SHapley Additive exPlanations (SHAP) analysis. Using SHAP analysis and BBO algorithm, we have found that hemoglobin and albumin mostly contribute to the detection of CKD.

## Introduction

Chronic kidney disease (CKD) is characterized by kidney damage or dysfunction measured by the Glomerular Filtration Rate (GFR). CKD is defined as the GFR less than 60 mL/min per 1.73 m^2^ for more than three months or markers of kidney damage^1–3^. The frequent causes of CKD are diabetes, hypertension, and other conditions^4,5^. The progression of CKD is silent; hence by the time an individual seeks medical care, they may already exhibit complications such as anaemia, cardiovascular disease, and nervous system disease^5,6^. The incidence of CKD grew by $89\%$ from $1990$ to $2016$, prevalence climbed by $87\%$, and deaths increased by $98\%$^7^. A study addressing the existing global nephrologists in $125$ United Nations member states (where $121$ countries answered the survey) revealed that the worldwide nephrologist density was $8.83$ Per Million Population (PPM)^8^. The study also shows the highest nephrologist density of $28.52$ PMP in high-income countries while the lowest nephrologist density of $.31$ PMP in underprivileged countries^8^. The International Society of Nephrology Global Kidney Health Atlas (ISN-GKHA) also reported that there are global shortages of nephrologists much greater in lower-income countries^8^. Hence it is much more necessary to develop an automatic diagnostic system to assist nephrologists in efficiently and accurately diagnosing a patient. The diagnostic system can be deployed in both low-income and high-income countries.

In recent decades many researches have been conducted to efficiently and accurately diagnose CKD patients. Taznin et al. conducted research on a CKD dataset and obtained $99\%$ accuracy using the Decision Tree (DT) algorithm taking only $15$ attributes out of $24$ features^9^. On the same CKD dataset, Amirgaliye et al. achieved about $94.60\%$ accuracy using a Support Vector Machine (SVM) classifier using all $24$ attributes^10^. Yildirim et al. identified CKD patients using Multilayer Perceptron (MLP) and a sampling technique, and they attained an F1 score of $99.8\%$^11^. Wibawa et al. combined K-Nearest Neighbor (KNN) with AdaBoost to classify CKD patients using $17$ attributes out of $24$ attributes that they selected using the Correlation-based Feature Selection (CFS) technique and achieved an accuracy of $98.1\%$^12^. Polat et al. achieved $98.5\%$ accuracy on an SVM classifier by employing the filter subset evaluator method as a feature selection (FS) strategy, which produced $13$ useful features out of $24$^13^. On the same dataset, Salekin et al. obtained a $99.3\%$ F1 score using a Random Forest Classifier (RFC) and also showed that a close result ($99\%$ F1 score) can be obtained using only ten relevant predictive attributes^14^. Manonmani et al. used the Improved Teacher Learner Based Optimization (ITLBO) algorithm as an FS technique that provided them with $16$ features out of $24$ and obtained an accuracy of $99.25\%$ using the Convolutional Neural Network (CNN) classification algorithm^15^. Rubini et al. used Fruit Fly Optimization Algorithm (FFOA) as an FS technique that resulted in $11$ relative attributes out of $25$ and obtained $99.08\%$ accuracy using Multi-Kernel Support Vector Machine (MKSVM) as the classifier^16^. On the same CKD dataset, Emon et al. tested several ML algorithms and found that RFC had the greatest accuracy $(99\%)$^17^. Gupta obtained $99.24\%$ accuracy in the same dataset using Logistic Regression (LR)^18^. Avci et al. classified the CKD using WEKA software and achieved $99\%$ accuracy with the $J48$ classifier^19^. Using the Multiclass Decision Forest (MDF) method, Gunarathne et al. decreased the attribute from $24$ to $14$ characteristics and achieved $99.1\%$ accuracy^20^. However, most of these studies lack an explanation of the ML model.

It should be mentioned that the majority of ML models are regarded as "black boxes". A black-box model is a sufficiently complicated model that can't be easily interpreted by humans^21,22^. When using the black box model as a diagnostic system, it is challenging for doctors to understand what caused the model to predict a specific result^23,24^. The black box approaches hinder medical decision support from physicians' and patients' perspectives^21^. Hence, it is necessary to develop a diagnostic system that provides the interpretability of the ML model. The interpretability of the ML model provides a safety check on the predicted results and increases the trust of the physicians in the system^21,23,25^. To address the interpretability of the ML model, the research interest in the field of eXplainable Artificial Intelligence (XAI) has been rising in recent years^26,27^. A survey on the XAI conducted by Amina et al. revealed a number of methods for deciphering ML models, including LIME, Decision Trees, Saliency Maps, Shapley Explanations, etc.^22,28–31^. In recent years, the SHapley Additive exPlanation (SHAP) has been used in various research to find the feature importance from the given set of features. Zhang et al. used SHAP to provide an interpretable model for Reinforcement Learning for Grid Control (RLGC)^32^. SHAP was utilized by Dikshit et al. to illustrate the significance of climatic factors impacting drought forecast^33^. Parsa et al. employed SHAP to examine the importance of traffic-related characteristics in the model that leads to more traffic accidents^34^.

Feature selection is a process of eliminating noisy features and selecting effective features from the dataset, which may improve the model's performance and prediction time^35,36^. Metaheuristic algorithms are frequently used in feature selection methods to obtain an optimum set of features^37,38^. Some of the well-known optimization algorithms are the Genetic Algorithm (GA), Particle Swarm Optimization algorithm (PSO), Biogeography based optimization (BBO), Ant Colony optimization algorithm (ACO), Whale Optimization Algorithm (WOA), etc.^39–43^. These types of algorithms can be used in ML to find the optimum feature set or to find an optimum parameter set for any ML model, etc.^44,45^. PSO-based feature selection was utilized by Sakri et al. to improve performance in predicting breast cancer recurrence^46^. In order to diagnose breast cancer, Alickovic et al. employed GA-based feature selection and got better results^47^. A comparison between GA, PSO, and BBO in finding an optimal feature set from Clusters of microcalcifications (MCC) conducted by Khehra et al. demonstrates that BBO performance is marginally superior to the other two^35,48^. In a CKD dataset, Manonmani et al. utilized the ITLBO method to find the best feature subset, and it chose 16 out of 24 features as the best feature subset^15^. The Correlation-based Feature Selection (CFS) approach was employed by Wibawa et al. on the same CKD dataset to identify the best features. The system chose 17 out of 24 characteristics as the best features^12^.

Motivating from these researches and findings, we have developed an automatic interpretable CKD diagnostic system that presents the priority of the attributes that influenced the system to diagnose a patient as either CKD or not CKD. The main contributions of this paper are, the CKD diagnostic system, an interpretable ML model through SHAP, and an analysis of the attribute contribution to the prediction of CKD. Also, BBO-based feature selection is used to obtain an optimum feature set, and SHAP is used to find their usefulness to the prediction. The paper is divided into several sections: “Methodology”, “Results”, “Discussions”, and “Conclusions”. The “Methodology” section is divided into subsections to discuss the dataset, SHAP, classifier, BBO, and performance metric in detail. In the “Results” section, we have sequentially presented our findings with the necessary data we obtained from the classifier, BBO, and the SHAP analysis. In the “Discussions” section, we have discussed the impact of the results and the impact of the research from a technical and medical science perspective. Finally, we have concluded and discussed future improvements of the research.

## Methodology

**Figure 1:**
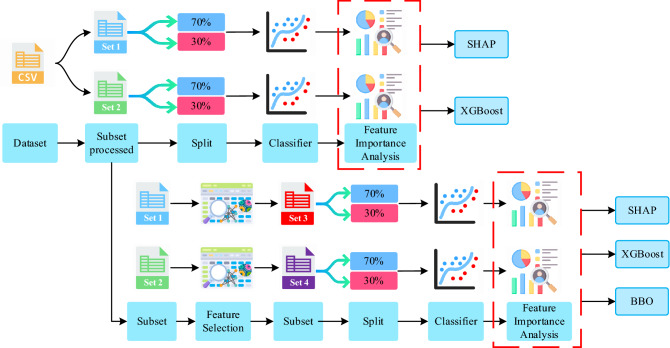
Working process.

We have developed a system to diagnose CKD and explain how the features influence the ML model to diagnose a patient into a particular class (CKD and not CKD). As the data in the real world can be messy and incomplete, we picked a similar dataset. In the "Dataset and preprocessing" section, we have discussed the dataset in detail. As the dataset is labeled (CKD, not CKD), our problem falls under the supervised ML problem. In Fig. 1, we have presented the working process of our work. First, we have created two subsets from the original dataset, namely "Set 1" and "Set 2". Later, we used the FS algorithm on the "Set 1" and "Set 2" subsets and made two new subsets, namely "Set 3" and "Set 4". Finally, we have classified the CKD on these four subsets separately and analyzed all these subsets using performance metrics and SHAP. To address the issues with the previous studies we have used SHAP-based feature importance to explain the ML model. In addition, we have used the BBO algorithm to further narrow down the essential features of the CKD dataset as shown in the figure. We have compared the SHAP feature importance with the XGBoost feature importance in the “Discussions” section to understand how each of them analyze the features.

### Dataset and preprocessing

**Figure 2:**
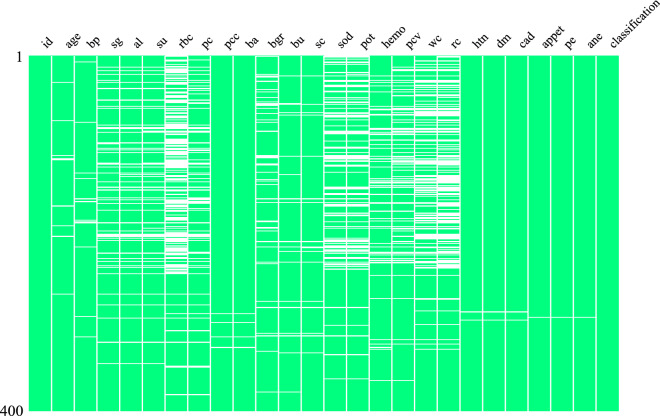
Visualization of missing values.

We have collected the CKD dataset from the University of California, Irvine (UCI) machine learning repository^49^. The dataset contains $24$ attributes of $400$ patients. Among the $400$ patients, $250$ are CKD patients, and $150$ are not CKD (NCKD) patients. The $24$ attributes, their description, and the missing value information are given in Table 1. Figure 2 shows a comprehensive overview of the missing values. From the table, we find most of the sample has a missing value of red blood cells, followed by red blood cell count and white blood cell count.

**Table 1:** Attribution of the CKD dataset.

Si no.	Name of the attribute	Abbreviation of the attribute	Value type	Number of missing values
1	Age	age	in years	$9$
2	Blood pressure	bp	mm/Hg	$12$
3	Specific gravity	sg	Nominal	$47$
4	Albumin	al	Nominal (1–5)	$46$
5	Sugar	su	Nominal (1–5)	$49$
6	Red blood cells	rbc	Normal, abnormal	$152$
7	Pus cell	pc	Normal, abnormal	$65$
8	Pus cell clumps	pcc	Present, not present	$4$
9	Bacteria	ba	Present, not present	$4$
10	Blood glucose random	bgr	mgs/dl	$44$
11	Blood urea	bu	mgs/dl	$19$
12	Serum creatinine	sc	mgs/dl	$17$
13	Sodium	sod	mEq/L	$87$
14	potassium	pot	mEq/L	$88$
15	Hemoglobin	hemo	gms	$52$
16	Packed cell volume	pcv	numerical	$70$
17	White blood cell count	wc	celles/cumm	$105$
18	Red blood cell count	rc	millions/cmm	$130$
19	Hypertension	htn	yes, no	$2$
20	Diabetes mellitus	dm	yes, no	$2$
21	Coronary artery disease	cad	yes, no	$2$
22	Appetite	appet	good, poor	$1$
23	Pedal edema	pe	yes, no	$1$
24	Anemia	ane	yes, no	$1$
25	Class	class	CKD, NCKD	$0$

The dataset contains many missing values that need to be processed. Depending on processing the missing values, we have created two different sets from the main dataset, namely "Set $1$" and "Set $2$". In "Set $1$", we have only kept the samples that did not contain any missing values. By these, the sample number was reduced to $158$ samples, among which $115$ are NCKD samples, and $43$ are CKD samples. In "Set $2$", we have replaced the missing value with the mean value; hence this set contains $400$ samples, among which $250$ are CKD patients, and $150$ are NCKD patients. We have split both sets into $70/30$ format. Here, 30% of the data is utilized for testing while 70% of the data is used for training. Later, in the “Discussions” section, we also presented the tenfold cross-validation results to better understand the stability of the model. Table 2 lists the number of training and testing samples.

**Table 2:** Sample sizes for training and testing set.

Name of the subset	Total sample	CKD	NCKD	Training	Testing
Set $1$	$158$	$43$	$115$	$110$	$48$
Set $2$	$400$	$250$	$150$	$280$	$120$

### Classifier

In this subsection, we have discussed the classifier we have used to classify the CKD dataset. A classifier is a kind of ML algorithm that categorized the data into predetermined classes. In this instance, the classifier will use the patient's characteristics as input to determine whether or not the patient has CKD.

In recent years the ensemble learning technique has become a popular choice for classification tasks^50^. Ensemble learning mainly has three classes: bagging, stacking, and boosting^51^. In the boosting technique, several weak learners are used together to build a strong classifier^50^. This type of classifier includes Gradient Boosting (GB), AdaBoost, XGBoost, etc.^50^. The GB employs a gradient-descending optimization algorithm to incorporate the weak learners in an effort to reduce the loss function. We have used XGBoost classifier to classify the CKD. The XGBoost classifier is an extension of the GB classifier, which also focuses on speed and performance^52^. The XGBoost includes the regularised learning that helps smooth the final learned weight to avoid overfitting^52^. The overfitting happens when the model starts to perform poorly on the testing data after learning the detail and noise from the training data. If we observe the objective function $L(\theta )$ of the XGBoostin Eq. (1) it includes a convex loss function and a penalty term,

1 $$L\left(\theta \right)=Loss \; term+Penalty \; term$$

2 $$L\left(\theta \right)= \sum_{i}l\left({y}_{pred}^{\left(t\right)}, {y}_{truth}\right)+ \sum_{k}\Omega \left({f}_{k}\right)$$

3 $$Where, \; \Omega \left({f}_{k}\right)=\Upsilon T+\frac{1}{2}\Upsilon {||\mathrm{w}||}^{2}$$

where $t$ is the training iteration, $l$ represents the training loss, which measures the difference between the predicted and actual values for each training instance. $k$ is the tree number, $T$ denotes the number of the leaf nodes, $w$ is the weight of each leaf, and $\lambda ,\Upsilon$ are the regularization term. The regularization parameters determine the relative penalty of each term which is used to avoid the overfitting by controlling the complexity^53–55^. Large weights are penalized by the regularization term, which also encourages the model to have more streamlined and comprehensible structural elements^56^. The goal of the model is to minimize this loss over the entire training set. Since our dataset is likewise noisy, XGBoost is an appropriate classifier for it. The values of the hyperparameters are given in the Table 3.

**Table 3:** XGBoost hyperparameter values.

Hyperparameter name	Value
eta (learning rate)	0.3
n_estimator (number of gradient-boosted trees)	100
Gamma (min split loss)	0
Max depth	6
Min child weight	1
Max delta step	0
subsample	1
Sampling method	Uniform

### Biogeography-based optimization algorithm

Feature selection is the process of finding a noise-free, effective set of features from a given dataset that improves the model’s performance on that dataset. The feature selection approach can be broadly divided into three main categories: embedded method, filter-based method, and wrapper-based method^57^. In the wrapper-based method, a subset of the total feature is evaluated using a ML algorithm iteratively to find the best feature subset^35^. Metaheuristic algorithms are mostly used in this case to iteratively find an optimum feature subset that provides maximum performance^58^.

BBO is a biology-based optimization algorithm introduced by Simon in $2008$^41^. Biogeography is a scientific approach to studying the geographical distribution of plants and species, which also concerns the factors responsible for the variation in distribution^59^. In BBO-based feature selection, each candidate solution or feature subset is called a habitat. Each potential solution is evaluated using an ML algorithm, which assigns a fitness score known as the Habitat Suitability Index (HSI)^41,59^. The habitat with high HIS has a high emigration rate and low immigration rate, while a habitat with low HIS has a high immigration rate and low emigration rate. The BBO mainly implements two functions, namely migration operation and mutation operation^41^. In migration operation, a relatively good solution tends to share its components with the other poor candidate solutions. This procedure is carried out for each element of a given solution, using the immigration rate of the solution as a probability. If the likelihood is in favor, that specific solution component is replaced by a component of a different solution selected from the population with a probability proportional to the emigration component of that solution. This selection process can be considered a roulette wheel selection. Since this process depends on probability, there is also a chance that the components of good solutions may immigrate and the components of poor solutions may emigrate. As in nature, any catastrophe can affect and change the habitat. This simulation process is called the mutation process, which changes the component of the habitat using a probability. Since the mutation process can change both the good and poor solution, the best solution may get lost. Hence an elitism approach is used to keep the best solution throughout the BBO process.

We have used the BBO algorithm to select an optimum feature subset from the 24 features of the CKD dataset. Here each habitat of the BBO represents a feature subset of the CKD dataset. The XGBoost algorithm is used as an evaluation function to evaluate the feature subsets. The number of gradient-boosted trees of the XGBoost was kept the same ($100$) throughout the optimization process. We have set the number of habitats and generation to $30$ and $50$, respectively. The mutation probability and the number of elite solutions were set to $.8$ and $2$, respectively. The BBO starts with the initial habitats, and the algorithm updates the habitats using the migration and mutation process. Finally, the BBO algorithms output an optimum habitat that is a feature subset that performed well on the evaluation function. By this, we have made two more subsets, namely "Set $3$" and "Set $4$" from "Set $1$" and "Set $2$" respectively.

### Performance metrics

**Figure 3:**
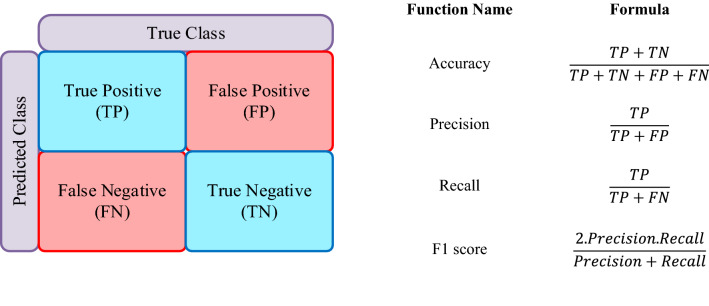
Confusion matrix and performance metrics formula.

Accuracy, Area Under Curve (AUC), precision, and recall are just a few of the measurement approaches used to gauge how well the classification ML model is performing. Also, the confusion matrix is the easiest and most intuitive metric used in the ML field. In Fig. 3, we have shown the confusion matrix along with the formula for various performance metrics.

The Receiver Operating Characteristic (ROC) curve has also been used to gauge the model's effectiveness. The ROC curve represents the relationship between the True Positive Rate (TPR) and False Positive Rate (FPR) at different threshold levels. To measure the classifier's ability to distinguish between classes, the Area Under the Curve (AUC) is used, whose value falls between zero to one. The model's performance improves with increasing AUC values. In addition, we have used the SHAP as the XAI tool to explain the model in depth. Shapley values are used by the SHAP to calculate the contribution of features based on its marginal contribution^60^. The SHAP analysis can be done over all the samples to find which features contribute most. Also, the SHAP can be used on a single sample which explains which features have influenced the model to predict a specific class and how much they influenced the model.

## Results

We divided the results into different subsections and presented them in this part. We have provided the classification result, Confusion Metrics, and ROC analysis in the "Classification results" section. In the "Explaining the model" section we have presented the SHAP analysis and discussed the influence of the features behind the prediction. Finally, we have presented a comparison table that shows the results obtained by others on the same CKD dataset.

### Classification results

From the data preprocessing stage, we have created two subsets from the original dataset. As the dataset contained missing values, we have created 'Set $1$' by dropping all the samples that had missing values. After removing the sample, the set contained only $158$ samples. Also, we have created another subset named 'Set $2$' where we replaced the missing values with the mean value. Hence 'Set $2$' contains $400$ samples. Further, to select the best feature set and reduce the model's complexity, we have used BBO algorithm. The BBO algorithm picked $9$ features from the "Set $1$" subset and $13$ features from the "Set $2$" subset. We have named the feature subset derived from "Set $1$" and "Set $2$" as "Set $3$" and "Set $4,$" respectively. Hence "Set $3$" contains nine attributes: hemoglobin, albumin, blood glucose random, coronary artery disease, diabetes mullites, potassium, blood urea, bacteria, and blood pressure as shown in Table 5. Whereas "Set $4$" contains $13$ attributes: albumin, pus cell clumps, bacteria, blood urea, sodium, potassium, hemoglobin, packed cell volume, white blood cell count, hypertension, diabetes mellitus, appetite, and anemia as shown in Table 5.

**Figure 4:**
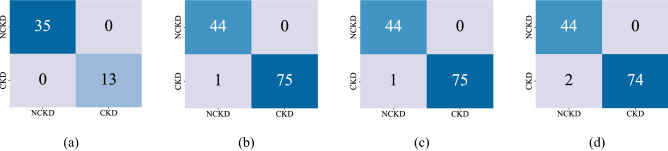
Confusion matrix of the testing set. (**a**) Set 1. (**b**) Set 2 (**c**) Set 3 (**d**) Set 4.

**Figure 5:**
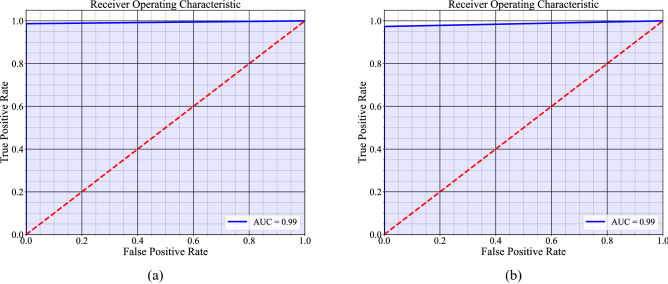
ROC analysis (**a**) Set 2. (**b**) Set 4.

These four subsets were evaluated separately using XGBoost classifier. The classification results and confusion matrix of all the sets are given in Table 4 and Fig. 4, respectively. The confusion matrix of "Set $1$" and "Set 3" presented in Fig. 4 shows that the model correctly classified all the samples. Whereas the model misclassified one sample from "Set 2" and two samples from the "Set $4$" subset. These total three samples were classified as NCKD while they belong to CKD class. We have traced these three samples to the original dataset and showed their attribute values are given in Table 5. We have analyzed their features in-depth in the "Explaining the model" section. The ROC analysis presented in Fig. 5 shows an AUC of $.99$ in both "Set $2$" and "Set $4$". The result on "Set 4" also shows that the model comparatively does well even with only $13$ features. The accuracy precision, recall, and F1 scores in "Set $4$" are $98.33\%, 100\%, 97.36\%,$ and $98.67\%,$ respectively. Whereas, in "Set $2$", the accuracy, precision, recall, and F1 scores are $99.16\%, 100\%, 98.68\%,$ and $99.33\%,$ respectively.

**Table 4:** Classification results on the CKD subsets.

Subset name	Train/test	Total sample	Feature number	Accuracy	Precision	Recall	F1 score
				%			
Removing all the samples that have missing values							
Set 1	Train	$110$	$24$	$100$	$100$	$100$	$100$
Set 1	Test	$48$	$24$	$100$	$100$	$100$	$100$
Replacing missing values with mean							
Set 2	Train	$280$	$24$	$100$	$100$	$100$	$100$
Set 2	Test	$120$	$24$	$99.16$	$100$	$98.68$	$99.33$
Using BBO algorithm on Set 1 we got Set 3							
Set 3	Train	$110$	$9$	$100$	$100$	$100$	$100$
Set 3	Test	$48$	$9$	$100$	$100$	$100$	$100$
Using BBO algorithm on Set 2 we got Set 4							
Set 4	Train	$280$	$13$	$100$	$100$	$100$	$100$
Set 4	Test	$120$	$13$	$98.33$	$100$	$97.36$	$98.67$

**Table 5:** Information on the selected features (with "Picked" denoting that the feature was chosen by the BBO algorithm and "–" denoting that it was not chosen) and misclassified samples.

Si no.	Name of the attribute	Abbreviation of the attribute	Set $3$	Set $4$	ID $0$	ID $140$	ID $173$
1	Age	age	–	–	$48$	$69$	$17$
2	Blood pressure	bp	Picked	–	$80$	$70$	$70$
3	Specific gravity	sg	–	–	$1.02$	$1.01$	$1.015$
4	Albumin	al	Picked	Picked	$1$	$0$	$1$
5	Sugar	su	–	–	$0$	$4$	$0$
6	Red blood cells	rbc	–	–	$0$	$0$	$0$
7	Pus cell	pc	–	–	$1$	$1$	$1$
8	Pus cell clumps	pcc	–	Picked	$0$	$0$	$0$
9	Bacteria	ba	Picked	Picked	$0$	$0$	$0$
10	Blood glucose random	bgr	Picked	–	$121$	$256$	$22$
11	Blood urea	bu	Picked	Picked	$36$	$40$	$1.5$
12	Serum creatinine	sc	–	–	$1.2$	$1.2$	$7.3$
13	Sodium	sod	–	Picked	$137$	$142$	$145$
14	Potassium	pot	Picked	Picked	$4$	$5.6$	$2.8$
15	Hemoglobin	hemo	Picked	Picked	$15.4$	$12$	$13.1$
16	Packed cell volume	pcv	–	Picked	$44$	$38$	$41$
17	White blood cell count	wc	–	Picked	$7800$	$8406$	$11200$
18	Red blood cell count	rc	–	–	$5.2$	$4$	$4$
19	Hypertension	htn	–	Picked	$1$	$0$	$0$
20	Diabetes mellitus	dm	Picked	picked	$1$	$0$	$0$
21	Coronary artery disease	cad	Picked	–	$0$	$0$	$0$
22	Appetite	appet	–	Picked	$1$	$1$	$1$
23	Pedal edema	pe	–	–	$0$	$0$	$0$
24	Anemia	ane	–	Picked	$0$	$0$	$0$
25	Class	class	CKD/NCKD	CKD/NCKD	CKD	CKD	CKD

### Explaining the model

**Figure 6:**
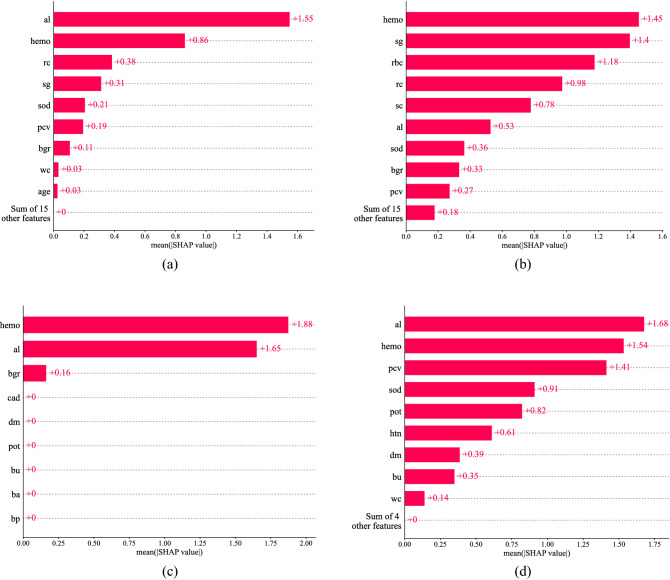
SHAP analysis on all samples. (**a**) Set 1. (**b**) Set 2. (**c**) Set 3. (**d**) Set 4.

The SHAP analysis of the four sub-sets is presented in Fig. 6. Figure 6 is called a global feature contribution plot as SHAP uses all the samples to calculate the SHAP values for each feature. Hence, the figure represents a generalized feature contribution ranking. The SHAP analysis of the model trained on "Set $1$" is displayed in Fig. 6a. The SHAP value is shown on the x-axis of the figure, while the feature rankings are shown on the y-axis. The features with higher SHAP values contribute most to detecting the class, hence are positioned on top. The features are sorted in the figure from high to low SHAP values. Figure 6a shows that albumin and hemoglobin have the highest SHAP value of $1.55$ and 0.$86$. It indicates that the model mostly used albumin and hemoglobin to predict CKD. But on "Set $2$", the analysis shows that hemoglobin, specific gravity, and red blood cells contributed most to detecting the CKD of $400$ samples. We can also find the SHAP analysis is a bit different in "Set $3$" and "Set $4$". In “Set $3$” among the nine features selected by the BBO algorithm, the SHAP analysis shows that hemoglobin, albumin, and blood glucose random contribute mostly to the CKD detection. Figure 6d shows that the albumin, hemoglobin, and packed cell volume mostly contribute to detecting CKD in the "Set $4$" subset. From the SHAP analysis we find, hemoglobin and albumin mostly contributed to the detection of CKD.

**Figure 7:**
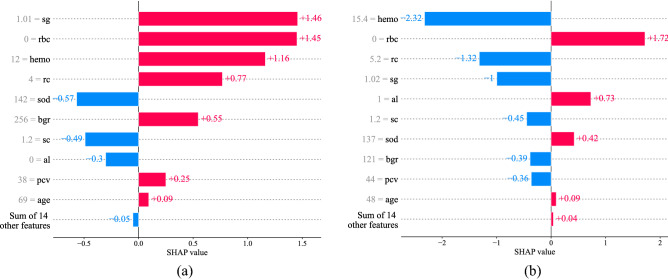
SHAP analysis of a single sample from Set 2. (**a**) Correctly classified (ID: 140, True: CKD, Predicted: CKD). (**b**) Misclassified (ID: 0, True: CKD, Predicted: NCKD).

**Figure 8:**
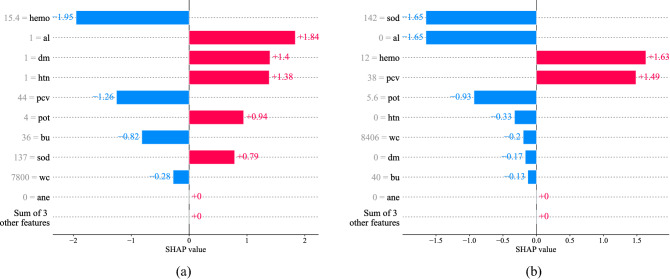
SHAP analysis of a single sample from Set 4. (**a**) Correctly classified (ID: 0, True: CKD, Predicted: CKD). (**b**) Misclassified (ID: 140, True: CKD, Predicted: NCKD).

We have also examined how a single sample's features impact the results. From the previous subsection, we find that the model misclassified one sample in "Set $2$" and two samples in "Set $4$". The ID of the misclassified sample in "Set $2$" is $0$, and the ID of the two other samples from "Set $4$" are $140$ and $173$. In Figs. 7 and 8, we have shown one correctly classified and one misclassified sample from both "Set $2$" and "Set $4$". For maximum comparison, we have taken ID $0$ and ID $140$ from both "Set $2$" and "Set $4$" as they were classified differently in both the subset. In "Set $2$", ID $140$ was classified correctly, and ID $0$ was misclassified as NCKD. While in "Set $4$", ID $0$ was classified correctly, but ID $140$ was misclassified as NCKD. The attribute values of these samples are shown in grey color on the left side of the features name.

In Figs. 7 and 8, the feature ranking is shown on the y-axis, while the x-axis displays the SHAP value. A feature can influence the model to predict a sample as CKD or NCKD. In the figure, the feature that affects the model to predict a sample as NCKD has a negative SHAP value and is shown in blue color. At the same time, the feature that influences the model to predict a sample as CKD has a positive SHAP value and is shown in red color. The SHAP value of each feature can be summed together to get the model's final output. If the output is positive, it indicates the model has identified the sample as CKD and if the output is negative, then the model has identified the sample as NCKD. From Fig. 7a, we find that the feature specific gravity, red blood cells, hemoglobin, packed cell volume, and age influenced the model to predict the sample as CKD. From the figure, we also find that the specific gravity and the red blood cells have the highest SHAP value hence, mostly influenced the model predicting it as a CKD sample. In contrast, we have also shown the SHAP analysis of an incorrectly classified sample in Fig. 7b. From Fig. 7b, we find that the features hemoglobin, red blood cell count, specific gravity, serum creatinine, blood glucose random, and packed cell volume have a negative SHAP value. Hence, they influenced the model to predict the sample as NCKD instead of CKD. If we summed the SHAP value of all the features, the resultant is a negative value. Hence the model finally outputs it as a NCKD sample. The hemoglobin level of 15.4 apparently influenced the model predicting the sample as NCKD. While in Fig. 7a (ID 140), the hemoglobin level was 12, which influenced the model toward CKD.

In the case of "Set $4$", ID $0$ was correctly classified, and ID $140$ was misclassified, whose SHAP analysis is presented in Fig. 8. Comparing the two SHAP analyses (Figs. 7b and 8a) of ID $0$, we find that red blood cell count, specific gravity, serum creatinine, and blood glucose random of "Set $2$" that influenced the model to predict it as NCKD are missing in "Set $4$". Again in the case of "Set $4$", the hemoglobin and packed cell volume strongly influence the model to predict the sample as NCKD followed by blood urea and white blood cell count. However, the average sum of the other features ultimately influenced the model predicting the sample as CKD. In the case of ID $140$, the feature specific gravity, red blood cells, red blood cell count, blood glucose random, and age of "Set $2$" that influenced the model to predict the sample as CKD are missing in "Set $4$". Even though the hemoglobin and packed cell volume of "Set $4$" are influencing the model to predict the sample as CKD, the negative contribution of the majority of the features makes the model predict the sample as NCKD. Hence the model misclassified the sample as NCKD.

### Comparison

In this subsection, we have presented the results of previous research on the same dataset in Table 6. From the table, we find N. Taznin et al., P. Yildirim et al., and A. Salekin et al. attained the dataset's best performance. Salekin et al. classified CKD with just ten characteristics and attained a 99% F1 score. Using the XGBoost, we have obtained an accuracy, precision, recall, and F1 score of $99.16\%, 100\%, 98.68\%,$ and $99.33\%,$ respectively, on "Set 2". Using the BBO feature selection technique, we found an optimal feature subset consisting only 13 features. On the "Set 4" subset, we have obtained accuracy, precision, recall, and F1 score of $98.33\%, 100\%, 97.36\%,$ and $98.67\%,$ respectively. Moreover, we have analyzed the feature contribution in both the subsets using SHAP and found that hemoglobin and albumin are the most influential attribute of CKD.

**Table 6:** Comparing results of other studies conducted on the same dataset.

Research conducted by	Classifier	Feature number	Metrics name	Metrics value (%)
Taznin et al.^9^	Decision tree	$15$	Accuracy	$99$
Amirgaliye et al.^10^	SVM	$24$	Accuracy	$94.6$
Yildirim et al.^11^	Multilayer perceptron with sampling algorithm	$24$	Precision	$99.8$
			Recall	$99.8$
			F1-score	$99.8$
Wibawa et al.^12^	KNN	$17$	Accuracy	$98.1$
			Precision	$98.0$
			Recall	$98.0$
			F1-score	$98.0$
Polat et al.^13^	SVM	$13$	Accuracy	$98.5$
Salekin et al.^14^	RFC	$10$	F1 score	$99.0$
Manonmani et al.^15^	CNN	$16$	Accuracy	$95.25$
			F1 score	$96$
Rubini et al.^16^	MKSVM	$11$	Accuracy	$98.5$
Emon et al.^17^	RF	$24$	Accuracy	$99$
Gupta et al.^18^	LR	24	Accuracy	$99.24$
			Precision	$98.82$
			Recall	$100$
Avci et al.^19^	J $48$	$24$	Accuracy	$99$
			Precision	$98$
			Recall	$99$
			F1-score	$98$
Gunarathne et al.^20^	MDF	14	Accuracy	$99.1$
Our result	XGBoost (without BBO) (Set 2)	$24$	Accuracy	$99.16$
			Precision	$100$
			Recall	$98.68$
			F1-score	$99.33$
	XGBoost (with BBO) (Set 4)	$13$	Accuracy	$98.33$
			Precision	$100$
			Recall	$97.36$
			F1-score	$98.67$

## Discussions

The obtained results on "Set $1$", "Set $2$", "Set $3$" and "Set $4$" presented in Table 4 indicate that CKD can be diagnosed quite accurately using ML. Using the BBO algorithm, we have reduced the number of features down to 9 ("Set 3") and $13$ (“Set 4”) features from the initial $24$ features. Analyzing all the models using the SHAP we find that hemoglobin and albumin are the most influential features in CKD diagnosis. Also, we have shown how the remaining features influence the model to predict CKD.

We have also used the tenfold cross-validation to find how well the model will perform on the unseen data. We have applied the cross-validation on all four of the subsets and collected their mean accuracy. The mean accuracy obtained in these four subsets is given in Table 7. The results suggest that the model is performing quite well in predicting CKD on unseen data. The result presented in Table 7 is also very close to the results of Table 4. From the results of Table 7, we have also calculated the p-value. Here Set $3$ is derived from Set $1$ and Set $4$ is derived from Set $2$ using the BBO algorithm. From the test results of Set $1$ and Set $3$, we have calculated the p-value of $1$ which is greater than alpha ($0.05$). This indicates that the results obtained using only $9$ features are quite similar to the results obtained using all $24$ features. Again from the test results of Set $2$ and Set $4$, we have calculated the p-value of $0.155$ which is greater than alpha ($0.05$). Thus the result we get using $13$ features are closer to the results we get using all $24$ features. We have reduced the model's complexity and obtained a state of the art results using almost half of the features. To further investigate how only a few of the initial features are mostly contributing to the model prediction we have used SHAP.

**Table 7:** Accuracy $(\%)$ result of the tenfold cross-validation obtained on the four subsets.

Name	Test 1	Test 2	Test 3	Test 4	Test 5	Test 6	Test 7	Test 8	Test 9	Test 10	Mean
Set $1$	$100$	$100$	$100$	$100$	$100$	$100$	$93.75$	$100$	$100$	$93.33$	$98.71$
Set $2$	$97.5$	$100$	$100$	$100$	$100$	$100$	$100$	$100$	$95.0$	$100$	$99.25$
Set $3$	$100$	$100$	$100$	$100$	$100$	$100$	$93.75$	$100$	$100$	$93.33$	$98.71$
Set $4$	$100$	$95.0$	$100$	$100$	$97.5$	$97.5$	$100$	$100$	$90.0$	$95.0$	$97.50$

Figures 6, 7, and 8, show how each feature influences the model to predict a certain class. The feature influence ranking can be used by a clinician to make clinical decisions more effectively. For instance, in Fig. 7a, the model is influenced by the sample’s specific gravity, red blood cells, and hemoglobin influence the model to predict the sample as CKD, whereas the model is influenced by the sample’s sodium level and serum creatinine to predict the sample as NCKD. This analysis gives an in-depth explanation of the model's prediction. A clinician can easily identify the contribution of each feature to each class and use them to make a rational decision or to tell the patient the reason behind their diagnosis. Moreover, the SHAP analysis also shows how much each feature influences the model to predict a class. This can help the clinician by indicating which attribute is most responsible for the detection of the patient's disease. In Fig. 7a, we find that the specific gravity, red blood cells, and hemoglobin mostly caused CKD detection in that particular patient as they have a higher SHAP value. Overall, the SHAP analysis of the ML model provides an extra layer of diagnosis that can be used by the clinician to be more certain about the diagnosis or to make a rational decision.

The XGBoost classifier also provides a feature importance ranking. We have gathered this feature importance on all four datasets to get a comparative result with the SHAP feature importance. The feature importance ranking obtained from the XGBoost classifier is presented in Table 8. If we compare the feature importance result of the XGBoost (Table 8) with the SHAP feature influence analysis (Table 9) we find that they don’t perfectly match. But if we look closely, we see that in Set $1$ the albumin has the highest ranking in both XGBoost and SHAP feature analysis. Again, in Set $2$, the hemoglobin has the highest ranking in both of the cases. In set $3$ both the albumin and hemoglobin are quite close to each other. In Set $4$, the hemoglobin is in the first rank in the XGBoost feature importance analysis and has the second rank in the SHAP feature analysis. Thus, we can say that both of the approaches are quite close to identifying the most influential features. In the case of XGBoost, it gives more weight to high cardinality features, while gain may be affected by tree structures. While in the case of SHAP, it uses game theory and estimates the impact of a feature on predictions. Finally, we can say that both of the feature importance methods indicate that hemoglobin, albumin, and a few others are the most influential features in the detection of CKD. Research conducted by other researchers also concluded that hemoglobin, albumin, and others are the top determinant of CKD detection^14,61^.

**Table 8:** XGBoost features importance on all four subsets.

Si no.	Set $1$	Set $2$	Set $3$	Set $4$
1	Albumin	Hemoglobin	Albumin	Hemoglobin
2	Red blood cell count	Red blood cell count	Hemoglobin	Diabetes mellitus
3	Hemoglobin	Specific gravity	Blood glucose random	Hypertension
4	Packed cell volume	Serum creatinine	–	Albumin
5	Specific gravity	Albumin	–	Packed cell volume
6	Sodium	Red blood cells	–	Potassium
7	Blood glucose random	Blood glucose random	–	Sodium
8	Age	Sodium	–	White blood cell count
9	White blood cell count	Blood pressure	–	Blood urea
10	–	White blood cell count	–	–
11	–	Packed cell volume	–	–
12	–	Blood urea	–	–
13	–	Age	–	–
14	–	Potassium	–	–

**Table 9:** SHAP features importance on all four subsets.

Si no.	Set $1$	Set $2$	Set $3$	Set $4$
1	Albumin	Hemoglobin	Hemoglobin	Albumin
2	Hemoglobin	Specific gravity	Albumin	Hemoglobin
3	Red blood cell count	Red blood cell	Blood glucose random	Packed cell volume
4	Specific gravity	Red blood cell count	–	Sodium
5	Sodium	Serum creatinine	–	Potassium
6	Packed cell volume	Albumin	–	Hypertention
7	Blood glucose random	Sodium	–	Diabetics mellitus
8	White blood cell count	Blood glucose random	–	Blood urea
9	Age	Packed cell volume	–	White blood cell count
10	–	Age	–	–
11	–	Potassium	–	–
12	–	Blood urea	–	–
13	–	White blood cell count	–	–
14	–	Blood pressure	–	–

The SHAP analysis can be used in other types of research where knowing the influence of features may play a major role. The XAI provides a way to examine the inner working of the ML model. This can be used to compare different ML model techniques to find how each ML technique uses the features to predict a particular class. In future work, different ML techniques can be explained using the SHAP to observe how each technique uses the feature to predict the class.

## Conclusions

Chronic kidney disease (CKD) is one of the major diseases among humans. Many countries lack experienced nephrologists and hence suffer more. Moreover, most of the existing ML models are considered black-box as they are very complex and don't provide how they output a certain prediction. In this paper, we have classified the CKD using XGBoost classifier. The BBO was used to reduce the feature number and attain an optimal feature subset. We have obtained accuracy, precision, recall, and F1 score of $99.16\%, 100\%, 98.68\%,$ and $99.33\%,$ respectively using all $24$ features. Also, we have obtained accuracy, precision, recall, and F1 score of $98.33\%, 100\%, 97.36\%,$ and $98.67\%,$ respectively using only $13$ features selected by the BBO algorithm. Analyzing the ML models trained on the original set and feature subset using SHAP, we find that hemoglobin and albumin largely influenced the model. Additionally, we see that the BBO algorithm also chose these attributes, along with a few additional traits, as the best features. We have demonstrated how each feature affects the model to classify a single sample for a given class. This analysis can aid in the clinician's decision-making and aid in the patient's understanding of the disease. This transparency of the black box ML model is beneficial to both the clinician and the patient. The system can be implemented in any hospital to aid an inexperienced nephrologist in making a more accurate diagnosis. Other, more sophisticated XAI methods may be employed in the future to explain the ML model.

## Data Availability

The dataset we have used and analyzed in our study is publicly available in the University of California, Irvine (UCI) machine learning repository (https://archive.ics.uci.edu/ml/datasets/chronic_kidney_disease). The codes and other data are available from the corresponding author and the first author upon reasonable request.
